# Early Motor Milestones in Infancy and Later Motor Impairments: A Population-Based Data Linkage Study

**DOI:** 10.3389/fpsyt.2022.809181

**Published:** 2022-01-31

**Authors:** Jing Hua, Gareth J. Williams, Hua Jin, Juan Chen, Manyun Xu, Yingchun Zhou, Guixiong Gu, Wenchong Du

**Affiliations:** ^1^Shanghai Key Laboratory of Maternal Fetal Medicine, Shanghai First Maternity and Infant Hospital, School of Medicine, Tongji University, Shanghai, China; ^2^School of Social Sciences, Nottingham Trent University, Nottingham, United Kingdom; ^3^Health Care Department of Suzhou Ninth People's Hospital, Suzhou, China; ^4^KLATASDS-MOE, School of Statistics, East China Normal University, Shanghai, China; ^5^Pediatrics Research Institution of Soochow University, Suzhou, China; ^6^NTU Psychology, Nottingham Trent University, Nottingham, United Kingdom

**Keywords:** motor impairment, developmental coordination disorder (DCD), crawling, independent walking, early motor milestone

## Abstract

**Background:**

Developmental Coordination Disorder (DCD) is a neurodevelopmental condition with high prevalence. Early motor milestones are important markers to identify DCD. The current study aims to evaluate the association between the onset of crawling and independent walking and their transition pattern during infancy and later motor impairments.

**Methods:**

A total of 8,395 children aged 3–6 years old in China were included in the final analysis. A parent questionnaire was used to collect early milestone onset data. Children's motor performance was measured using the Movement Assessment Battery for Children-2nd edition (MABC-2). The association between motor milestones and motor impairment was analyzed using a multilevel regression model.

**Results:**

The result showed that a 1-month delay in crawling onset increased the risk of significant overall motor impairment by 5.3, and 14.0% when adjusting for child and family characteristics. A 1-month delay in walking onset increased the risk of significant overall motor, fine, gross, and balance impairment by 21.7, 8.3, 13.3, and 17.8%. A 1 month increase in the transition time from crawling to independent walking increased the risk of significant overall motor and gross motor impairment by 7.7 and 6.6%. These results were inconsistent across different age bands (each *p* < 0.05).

**Conclusions:**

Our study indicates that even a mild delay in crawling and walking onsets in infancy increase the risk for subsequent motor impairments in childhood, and children with motor impairments revealed a different transition pattern from crawling to walking. The motor abilities of children with motor impairments can be observed to diverge from typically developing children as early as 6–8 months old. The findings can facilitate the early identification of motor impairments in children, and provide early signs to initiate intervention.

## Introduction

Developmental Coordination Disorder (DCD) is a neurodevelopmental condition marked by impairments of motor coordination. Studies have shown that the prevalence of DCD is around 5–6% in school-aged children (1, 2). One of the diagnostic criteria of DCD emphasizes that symptoms of DCD can be observed from early childhood (3, 4), and early intervention can help to reduce the emotional, physical, and social consequences that are often associated with this disorder (5, 6). Therefore, it is important to understand what early signs in motor development are associated with later motor impairments, when motor abilities of children with DCD start to diverge from typical developmental children, and to what extent the delays in motor development can facilitate the early identification of DCD.

During infancy, motor development is manifested at the behavioral level as a progression of new motor milestones (e.g., crawling, walking, etc.). Delayed motor milestone onsets during infancy can reflect a delay in physical and neurological development, and therefore is an important early identifier of childhood developmental disorders (7). For children with DCD, however, very few studies have specifically focused on that timeframe of motor milestones (8, 9). Findings, with a small sample, indicate that only children with DCD but not children with Autism reach key motor milestones significantly later than typically developed children (10). Moreover, motor milestones have been suggested as a potential early symptom of DCD by DSM-5 (3). However, there was a lack of consistent data on whether there is an association between the motor milestone delays and DCD (11), and to what extent the delays in motor milestones can aid in the early identification of motor impairments.

Additionally, motor development requires peripheral and biomechanical readiness for engendering developmental change (12). The change from crawling to walking is one of the most significant examples of this process. As a qualitative change in development, the transition reflects the rate of change in body control and coordination skills. Most infants start to walk independently after crawling (13), and the transition from crawling to walking spans several weeks. However, such developmental trajectory data are lacking in DCD. To our knowledge, the only relevant data in the literature did not find the transition time from crawling to walking is different between children with DCD and controls, but the small sample size of the study makes it hard to draw a reliable conclusion (10). It is therefore unclear whether children with DCD have a different developmental pattern in their transition from crawling to walking compared to typically developing children, and to what extent the pattern can facilitate the early identification of DCD in infancy.

Therefore, in this study, we conducted a population-based study to explore a longitudinal association between early motor milestones and motor impairment reported at a later age (3–6 years old). We hypothesized that DCD children have a delayed onset of motor milestones and may also develop at a slower rate compared to their typically developing peers. Therefore, the objectives of the current study were: firstly to evaluate to what extent the onset of crawling and walking in early infancy is associated with later motor impairment, and secondly whether children with motor impairment follow a similar developmental transition from crawling to walking as typically developing children.

## Methods

### Study Design and Population

**Figure 1:**
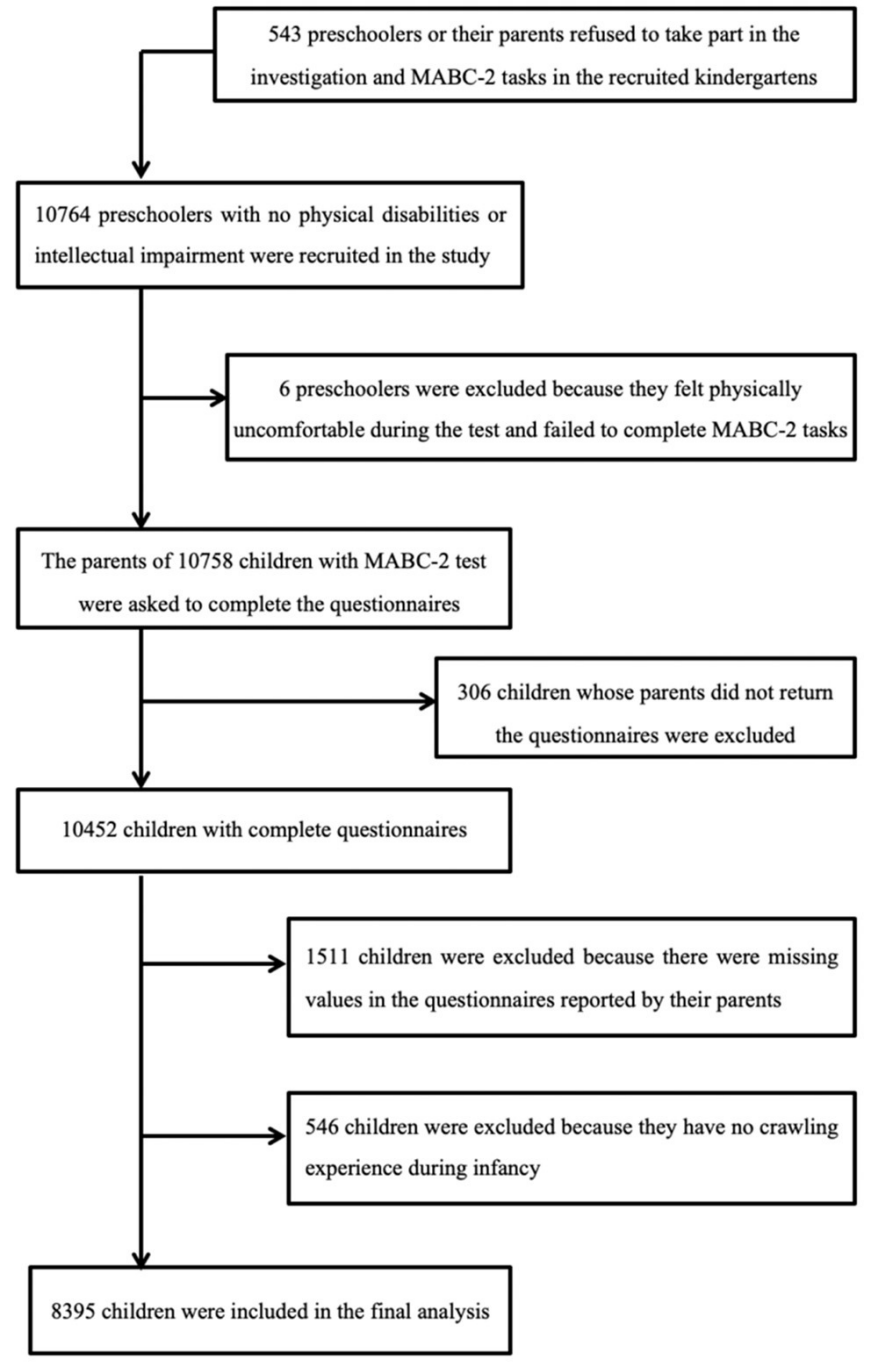
Flowchart of the study population.

We conducted a population-based data linkage study in 5 cities in China between March 1st 2010 and January 31st 2012. The database was derived from data held by the city's regional children's healthcare institution, which collects data on children's physical status and motor milestones during infancy and toddlerhood. Using this database as the base population, we carried out follow-up data collection with these children when they were preschoolers. According to the local health and education authority regulations, children typically visit the children's health care institution and enter kindergarten in the same district of the city. Only mainstream schools and nurseries were included in the study. Children with severe visual, hearing, intellectual impairments (according to the examinations before starting kindergarten), or other severe developmental disorders who were required to attend special education schools/nurseries according to the local regulations were excluded. A total of 10,758 children were assessed by 42 assessors with the standardized motor assessment. All assessors were qualified child therapists and were trained to administrate assessment. The interrater reliability was demonstrated to be good (14). Class teachers were responsible for distributing the notification to parents to complete the questionnaire; names and phone numbers of the researchers were provided in case the parents had queries. Parents of 10,452 children completed and returned the questionnaires concerning their child and family characteristics after giving consent to participate in the study. Each child's motor scores, responses to the child's parental questionnaire, and their base data during infancy were record-linked to conduct the analysis. Of the initial dataset, 8,395 children were included in the final analysis (Figure 1). Records were removed where there was missing infancy data or where an infant had not reported having crawled. The study was approved by the local Education Board and Ethics Committee of the Children's Hospital of Soochow University. Parental consent and children's assent were obtained before testing. All information acquired was kept confidential and was accessible only to the researchers.

### Measures

#### Outcomes

Children's motor impairments were assessed using the Movement Assessment Battery for Children-2nd edition (MABC-2) (15), which is a diagnostic measurement for DCD in childhood. The age band 1 of the MABC-2 test, which refers to children aged 3–6 years old, was used in our study. The test contains eight items categorized into the following three motor subtests (manual dexterity, aiming and catching, and balance). The scores of subtests are added up by their corresponding items. The total score of the MABC-2 is the sum of the standard scores for all eight items. The better a child's performance, the higher the test score. According to the previous research (14), MABC-2 is a valid and reliable measurement when it is used with Chinese children. However, because it is not generally recommended to make a diagnosis on children based only on a diagnostic test (16), we used the term “motor impairment” instead of DCD. We grouped them (aged 3–6 years old) into significant motor impairment (≤ 5th percentile of the total score), at-risk of motor impairment (6–16 percentiles of the total or subtest score) and typical motor performance (>15 percentile of the total or subtests' score) according to MABC-2 Examiner's Manual (15). The procedure for conducting the MABC-2 has been described in previous studies (17, 18).

#### Predictors

Crawling is defined as when a child alternatively moves forwards or backwards in a four-point position on hands and knees with the abdomen off the floor, with arms and legs moving reciprocally (19, 20). Independent walking is defined as walking without assistance according to the World Health Organization (WHO) motor development study (19). We asked parents “how old was your child in months when she or he started to move forward or backward on BOTH hands and BOTH knees without the abdomen contacting the floor?” with regard to crawling; and “how old was your child in months when she or he started to walk independently” with regard to independent walking. We used the time difference between crawling and independent walking to describe the transition time from crawling to walking. In preterm children, we used a corrected month age of crawling and walking onset. Children who did not experience hands-and-knees crawling, but presented bottom shuffling or bunny-hopping during infancy which might be related to more complex mechanisms, were excluded from our study.

#### Covariates

Body Mass Index (BMI) is an indicator of obesity that is based on height and weight (BMI = weight (kg)/height (m) according to the WHO BMI classification (21). Family structures were classified into three types: “three-generation (or more) family,” “nuclear family,” and “single-family.” The “three-generation (or more) family” type refers to a child living with his/her parents and grandparents, and is a traditional family structure in China. “Nuclear family” refers to where a child lives with their parents, and “single mother or father” means the child lives with one of their parents. All covariates are shown in Table 1.

**Table 1:** The children's and family's characteristics (*n* = 8,395)^a^.

**Characteristics**	**Significant motor impairment (≤5th centile of MABC-2)**	**At-risk motor impairment (6~16th centile of MABC-2)**	**Typical performance (>16th centile of MABC-2)**
**CHILD CHARACTERISTICS**			
Children's age (*n*%)			
3	50 (10.6)	118 (12.5)	742 (10.6)
4	141 (29.8)	280 (29.6)	2,138 (30.6)
5	147 (31.1)	339 (35.8)	2,336 (33.5)
6	135 (28.5)	209 (22.1)	1,760 (25.2)
Sex			
Male	219 (46.3)	442 (46.7)	3,142 (45.0)
Female	254 (53.7)	504 (53.3)	3,834 (55.0)
BMI (*n*%)^***^			
≤ 18	421 (89.0)	895 (94.6)	6,436 (92.3)
>18	52 (11.0)	51 (5.4)	540 (7.7)
Right handedness (*n*%)			
No	23 (4.9)	50 (5.3)	282 (4.0)
Yes	450 (95.1)	896 (94.7)	6,694 (96.0)
Eye-sight^b^			
Normal	454 (96.0)	877 (92.7)	6,538 (93.7)
Abnormal	19 (4.0)	69 (7.3)	438 (6.3)
Gestational weeks (*n*%)			
<37	23 (4.9)	64 (6.8)	521 (7.5)
≥37	450 (95.1)	882 (93.2)	6,455 (92.5)
Birth weight (*n*%)			
<2,500 g	22 (4.7)	35 (3.7)	340 (4.9)
≥2,500 g	451 (95.3)	911 (96.3)	6,636 (95.1)
**FAMILY CHARACTERISTICS**			
Mother has a higher education (*n*%) ^**^			
No	198 (41.9)	412 (43.6)	3,323 (47.6)
Yes	275 (58.1)	534 (56.4)	3,653 (52.4)
Father has a higher education (*n*%) ^**^			
No	163 (34.5)	334 (35.3)	2,789 (40.0)
Yes	310 (65.5)	612 (64.7)	4,187 (60.0)
Family annual per-capita income (RMB) (*n*%) ^c^^***^			
Below	303 (64.1)	657 (69.5)	5,316 (76.2)
Above or equal to	170 (35.9)	289 (30.5)	1,660 (23.8)
Family structure (*n*%)			
Single families	6 (1.3)	19 (2.0)	102 (1.5)
Nuclear families	291 (61.5)	589 (62.3)	4,530 (64.9)
Extended families	176 (37.2)	338 (35.7)	2,344 (33.6)
The number of children in the family (*n*%)			
One	386 (81.6)	761 (80.4)	5,449 (78.1)
Two or more	87 (18.4)	185 (19.6)	1,527 (21.9)
Maternal age at delivery (*n*%)			
<30	396 (83.7)	803 (84.9)	5,898 (84.5)
30-34	64 (13.5)	114 (12.1)	821 (11.8)
≥35	13 (2.7)	29 (3.1)	257 (3.7)
Maternal complications during pregnancy^d^ (*n*%)^**^			
No	367 (77.6)	797 (84.2)	5,650 (81.0)
Yes	106 (22.4)	149 (15.8)	1,326 (19.0)

a *Pearson chi-square test.*

b *Eye-sight refer to the children's visual acuity, which is tested by reading a Snellen eye chart at a distance of 20 feet (A normal eye-sight menas when you stand 20 feet away from the chart you can see what a “normal” human being can see at 20 feet).*

c *The national average family per-capita income of the year before the survey time.*

d *Having one of the following maternal complications during pregnancy including vaginal bleeding during pregnancy, at risk of miscarriage, use of antibiotics, use of fertility drugs, intrauterine distress, fetal asphyxia.*

** *p < 0.01,*

*** *p < 0.001*.

#### Statistical Analyses

Chi-square analyses were used to test for significance in comparing children's age, sex, BMI, parents' education, and family income between children with motor impairments and with typical motor performance. In order to assess the effects of crawling and walking onset, and the transition from crawling to walking on children's motor impairments (0 = typical performance, 1 = at-risk of motor impairment, 2 = significant motor impairment), odds ratios were estimated to determine the strength of the association using a multilevel logistic regression model. In this model, we utilized a random intercept (we considered the kindergarten as a cluster, and hypothesized that there was no interaction between kindergartens and gross motor milestones) to investigate the associations between motor milestone and risk of motor impairment when adjusting for the clustering (kindergartens) and other potential confounders (child and family characteristics which are presented in Table 1). Additionally, we conducted a stratified analysis in children aged 3–4 and 5–6 years old. All analyses were performed in R 2.15.1 using the MGCV and LME4 packages. *p* < 0.05 was considered statistically significant.

## Results

Of the 8,395 children included in the final analysis, the mean month of crawling and independent walking onset was 8.1 and 12.6 months, with a standard deviation (SD) of 1.8 and 1.7 months, respectively. The total score and motor subtest scores of the MABC-2, comparing children and family characteristics are shown in Supplementary Table 1. The child and family characteristics by motor impairments were shown in Table 1 and Supplementary Table 2.

The results showed that a 1 month delay in crawling onset increased the risk of overall significant motor impairment by 5.3% (as measured by the total MABC-2 score) and 4.7% for at-risk motor impairment when compared to typically developing children and adjusting for month age of independent walking and child and family characteristics (each *p* < 0.05). There was also a statistically significant, 14.0% (significant group) and 7.9% (at-risk group) increased risk of balance impairment (each *p* < 0.05) when adjusting for the same characteristics. The crude and adjusted OR with 95% CI are presented in Table 2.

**Table 2:** Effects of timing crawling and walking during infancy on motor impairments (*n* = 8,395).

**Characteristic**	**Significant motor impairment vs. typical performance (≤5th centile of MABC-2)** **(>16th centile of MABC-2)**			**At-risk motor impairment vs. typical performance (6~16th centile of MABC-2)** **(>16th centile of MABC-2)**		
	**cOR^a^ (95% CI)**	**aOR (95% CI)**	**aOR (95% CI)**	**cOR (95% CI)**	**aOR (95% CI)**	**aOR (95% CI)**
**OVERALL**						
Onset of crawling (age month)	1.051 (1.000, 1.104)^a^^*^	1.015 (0.963, 1.068)^b^	1.053 (1.000, 1.107)^d^^*^	1.045 (1.007, 1.083)^a^^*^	1.024 (0.986, 1.063)^b^	1.047 (1.008, 1.086)^d^^*^
Onset of independent walking (age month)	1.214 (1.146, 1.286)^a^^*^	1.210 (1.14, 1.283)^c^^***^	1.217 (1.148, 1.290)^e^^***^	1.118 (1.072, 1.167)^a^^***^	1.112 (1.064, 1.162)^c^^***^	1.119 (1.072, 1.168)^e^^***^
Time difference between crawling and walking (age month)	1.075 (1.029, 1.125)^a^^***^	—	1.077 (1.030, 1.126)^f^^**^	1.029 (0.996, 1.063)^a^	—	1.028 (0.996, 1.062)^f^
**FINE MOTOR**						
Onset of crawling (age month)	1.024 (0.973, 1.076)^a^	1.009 (0.957, 1.062)^b^	1.026 (0.977, 1.077)^d^	1.009 (0.972, 1.047)^a^	1.002 (0.964, 1.041)^b^	1.010 (0.973, 1.048)^d^
Onset of independent walking (age month)	1.081 (1.019, 1.146)^a^^**^	1.078 (1.015, 1.145)^c^^*^	1.083 (1.020, 1.148)^e^^**^	1.035 (0.991, 1.081)^a^	1.035 (0.99, 1.081)^c^	1.036 (0.992, 1.082)^e^
Time difference between crawling and walking (age month)	1.026 (0.981, 1.072)^a^	—	1.027 (0.981, 1.073)^f^	1.013 (.980, 1.046)^a^	—	1.012 (0.981, 1.046)^f^
**GROSS MOTOR**						
Onset of crawling (age month)	1.008 (0.965, 1.052)^a^	0.983 (0.94, 1.027)^b^	1.010 (0.968, 1.055)^d^	1.026 (0.988, 1.064)^a^	1.012 (0.974, 1.05)^b^	1.028 (0.990, 1.066)^d^
Onset of independent walking (age month)	1.131 (1.077, 1.188)^a^^***^	1.136 (1.08, 1.195)^c^^***^	1.133 (1.077, 1.189)^e^^***^	1.078 (1.033, 1.126)^a^^**^	1.075 (1.029, 1.123)^c^^**^	1.079 (1.034, 1.127)^e^^*^
Time difference between crawling and walking (age month)	1.067 (1.027, 1.108)^a^^***^	—	1.066 (1.026, 1.108)^f^^***^	1.023 (0.990, 1.056)^a^	—	1.022 (0.990, 1.057)^f^
**BALANCE**						
Onset of crawling (age month)	1.139 (1.084, 1.196)^a^^***^	1.111 (1.055, 1.169)^b^^***^	1.140 (1.084, 1.197)^d^^***^	1.078 (1.040, 1.116)^a^^***^	1.063 (1.025, 1.102)^b^ ^***^	1.079 (1.05, 1.117)^d^^***^
Onset of independent walking (age month)	1.176 (1.107, 1.249)^a^^***^	1.144 (1.075, 1.218)^c^^***^	1.178 (1.109, 1.251)^e^^***^	1.092 (1.047, 1.139)^a^ ^***^	1.075 (1.03, 1.123)^c^ ^***^	1.093 (1.048, 1.140)^e^^***^
Time difference between crawling and walking (age month)	0.987 (0.942, 1.034)^a^	—	0.986 (0.941, 1.034)^f^	0.992 (0.961, 1.024)^a^	—	0.991 (0.960, 1.024)^f^

a *Not adjusted for other variables*.

b *Adjusted for month age of independent walking onset.*

c *Adjusted for month age of crawling onset.*

d *Adjusted for month age of independent walking onset and children's and family's characteristics (all covariates in Table 1).*

e *Adjusted for month age of crawling onset children's and family's characteristics (children's age, sex, BMI, parents' education, and family income) (all covariates in Table 1).*

f *Adjusted for children's and family's characteristics (children's age, sex, BMI, parents' education, and family income).*

* *p < 0.05,*

** *p < 0.01,*

*** *p < 0.001*.

Compared with typically developing children, a 1 month delay in the onset of independent walking increased the risk of overall motor impairment by 21.7% for the significant motor impairment group; by 11.9% for the at-risk of motor impairment group; by 8.3% for significant fine motor impairment group; by 13.3 and 7.9% for the significant and at-risk of gross motor impairments when adjusting for month age of crawling and child and family characteristics (each *p* < 0.05). A 1 month delay in the onset of independent walking increased the adjusted risk of balance impairment by 17.8% for the significant motor impairment group, and 9.3% for the at-risk of motor impairment group (each *p* < 0.05). The crude and adjusted OR and 95% CI are shown in Table 2.

We found that a 1 month increase in transition time from crawling to independent walking increased the risk of overall significant motor impairments by 7.7% when compared with the typically developing group, and adjusting for child and family characteristics. An increase in timing difference between crawling and independent walking was significantly associated with subsequent significant impairment in the gross motor subtest when adjusting for child and family characteristics (OR = 1.066, *p* < 0.05). The crude and adjusted OR and 95%CI are shown in Table 2.

**Figure 2:**
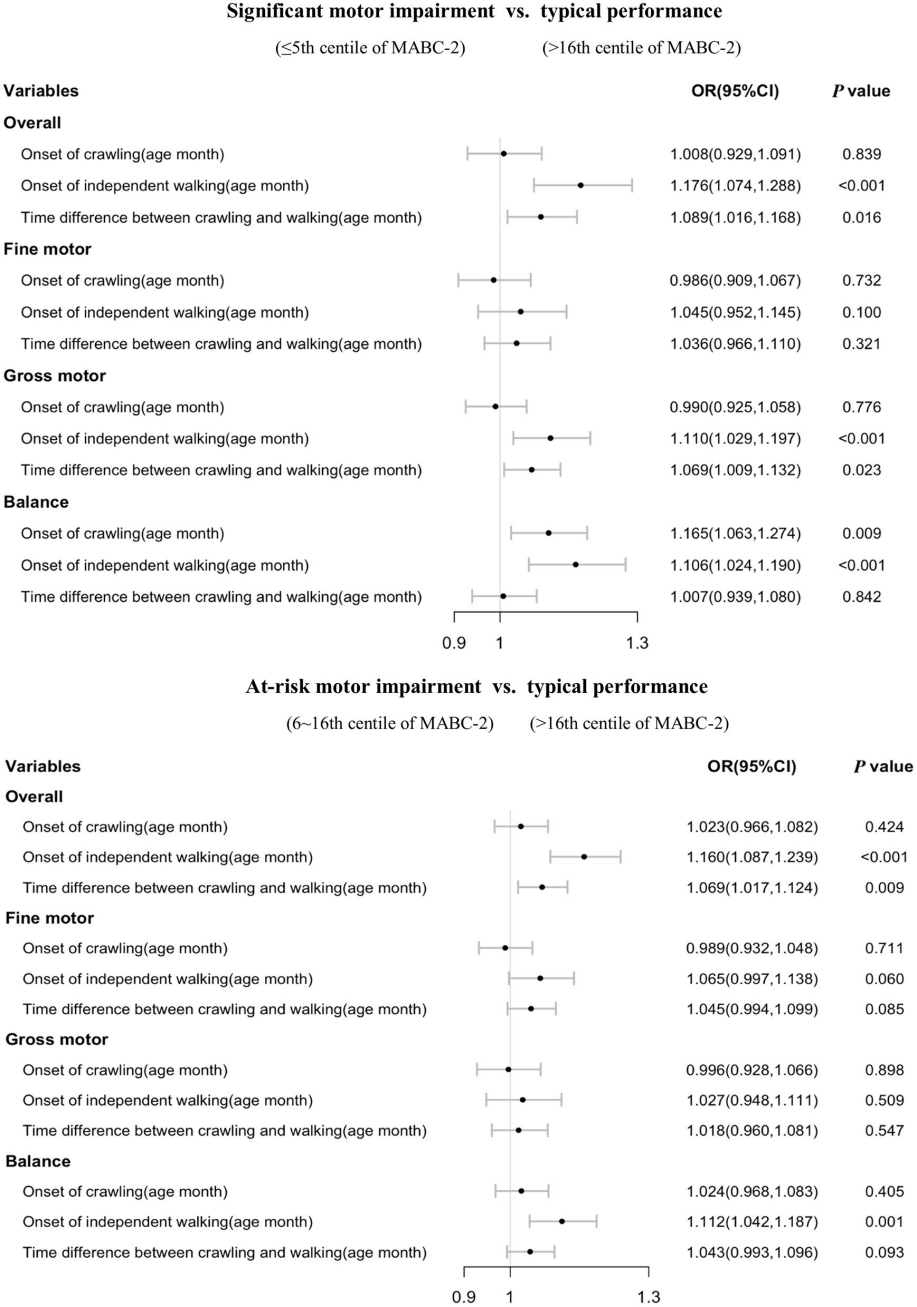
Association of walking and crawling during infancy with motor impairments by different age group in children aged 3–4 years old (*n* = 3,469).

**Figure 3:**
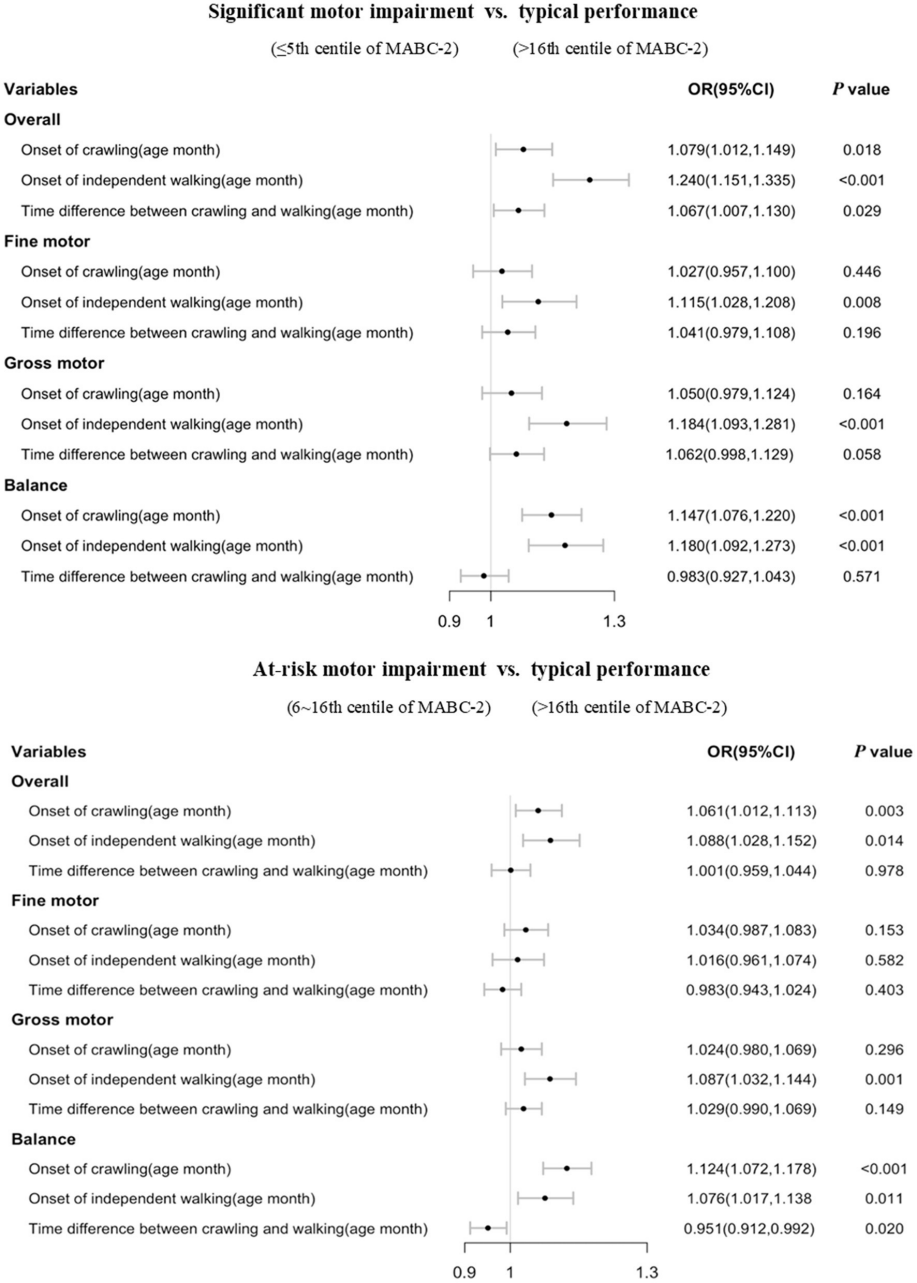
Association of walking and crawling during infancy with DCD by different age group in children aged 5–6 years old (*n* = 4,926).

Stratified analysis showed that most of the significant associations of crawling and independent walking with later motor impairment remained in children aged 3–4 years old (each *p* < 0.05, Figure 2), however, some of the associations between motor milestones and at-risk of motor impairment disappeared in children aged 5–6 years old (Figure 3). We found that the time difference between crawling and walking could predict significant overall motor impairment and gross motor impairment in children aged both 3–4 and 5–6 years old (each *p* < 0.05), but could not be considered as predictors for the at-risk motor impairments (each *p* > 0.05).

## Discussion

To our knowledge, our study is the first population-based study that explored a longitudinal association between early motor milestones and the impairment of motor coordination revealed at a later age (3–6 years old), and our results confirmed that a delayed onset time of crawling and independent walking is associated with subsequent motor impairment. More importantly, our results also suggested that a long transition time from crawling to walking is associated with subsequent childhood motor impairments, with children with motor impairment having a longer transition time between crawling and independent walking when compared with typically developing children. Our study provided quantitative information to assist our early identification of children with motor impairments.

Our results revealed a similar prevalence of children with significant motor impairments (5.2–6.4%) to previous reports (16, 22). More importantly, our results demonstrated a relatively consistent result that the late crawling onset time is associated with balance impairment at a later stage. As early as 6–8 months, the motor abilities of children with DCD can be observed to diverge from typically developing children by having a delayed crawling onset, which mostly reflects an impaired balance development at a later stage. Neurological evidence has shown that crawling experience is accompanied by neural changes, leading to a more efficient cortical organization (23), which, however, has been found altered in children with DCD (24–26). Crawling requires the coordination of a range of different muscles in the infant's trunk and lower extremities, and the interlimb coordination of overall balance is a key factor in an infant's transition from belly crawling to hands-and-knees crawling (27). The process of crawling provides novel eye-hand coordination, vestibular processing, tactile input, and kinesthetic awareness experiences, which are essential to the integration of sensory and motor systems and the improvement of balance (28). This is also in line with our results that a delayed crawling onset was only associated with impairment in balance but not fine or gross motor skills.

The timing of walking onset was a more powerful predictor compared to the crawling onset on motor impairments, and walking onset can predict children's later fine, gross, and balance motor impairments. Although each gross motor milestone in infancy has both similar and different components, independent walking is probably the synthesis of all these components (29, 30). The coordination of muscles in the trunk and lower extremities, along with reciprocation between muscles and balance is needed to achieve walking (29, 30). Although previous reports of the association between early gross motor milestones and later fine motor development are mixed (8, 31), the theoretical background in occupational therapy suggests that a reduction in high level tactile, proprioceptive, and kinesthetic input affects the quality of upper extremity functioning (32, 33). Therefore, as shown in this study, delayed onset of independent walking is also related to manual dexterity impairments in the group with significant motor impairment. These findings demonstrate a consistent pattern of delayed motor development in infancy is associated with later motor impairment and early motor milestone onset might assist the identification of DCD.

The most novel finding of our study was that 1 month increase in the transition time between crawling and walking can increase the risk of overall motor impairment. The inconsistent developmental pace has been found in other developmental disorders. For example, children with autism have been reported to have heterogeneous developmental pathways, with evidenced remarkable developmental change over time (34–36). Most infants with motor impairment seem to suffer a developmental delay in early motor skill achievement but can eventually catch up and reach the next motor milestone at a similar pace. It is possible that children with DCD crawl longer before starting to walk because they require more practice by crawling to be peripherally and biomechanically ready for independent walking. Slowed rate of growth may index aberrant processes during early development and precede the onset of symptoms. It should also be noticed that the delayed transition time between crawling and walking was only associated with significant motor impairment but not at risk of motor impairment, and its association with significant motor impairment seems to mainly reflect a subsequent impairment in gross motor skills. The results further suggested that the transition from crawling to walking is a complex process and compared to children with mildly delayed motor development, children with severe motor impairment or DCD may suffer from issues in more than the development rate of one group of motor capabilities; and different developmental patterns may exist between children with DCD and children with mildly delayed motor development. In this case, a developmental trajectory approach may be needed in future research to examine the differences in developmental patterns of children with DCD (i.e., more severe motor impairment) and children with mildly delayed motor development, as well as typically developed children (37). A focus on the developmental pattern can allow further factors to be studied beyond delay, which may ultimately index different underlying developmental pathways of DCD.

Additionally, we observed inconsistent results across different age bands in our stratified analysis. Most of the significant associations between motor milestones onset time and later motor impairment remained in younger children aged 3–4 years old, however, some of the associations disappeared in older children (aged 5–6 years old). It has been reported that early motor experiences can affect subsequent motor development (38). Previous studies have found environmental risk factors in kindergartens and families that were associated with childhood motor impairment (17, 22). Therefore, the association between the onset time of motor milestones and motor impairment might also be related to the positive or negative impact of a child's environment. Further study is needed to explore the mechanism of age differences in the pattern of associations.

There were several limitations of the current study. First, we should consider the possibility that other conditions such as undiagnosed attention problems or other undiagnosed psychological or neuropsychological impairments may affect the children's performance in the MABC-2 test; and not all children with poor movement performance as defined by the MABC-2 test would be clinically diagnosed as DCD. However, the focus of the current study was the association of early motor milestones and subsequent motor impairments, and future studies should be conducted to further explore the potential different developmental trajectories between DCD and other neuropsychological disorders that often have motor impairment as a symptom. Secondly, we used the onset time of both crawling and walking reported by the parents as the indicator of the children's early motor development. It is possible that reports by parents are inaccurate. However, an important strength of the present study is that it was based on a larger sample than those previously described and that a wide range of confounding variables including child and family characteristics have been measured and controlled for in our analysis. A large population-based sample reduces the possible errors in parents' estimated reports regarding the children's motor development history and attenuates the variation of motor activity that might occur in infancy. Moreover, motor impairment is a minor condition compared to more severe conditions such as cerebral palsy or Down Syndrome. Most parents do not have knowledge of DCD (39) and children with DCD are rarely diagnosed (40) especially in China where the study was conducted. Therefore, the parents of children with and without motor impairments as defined in the current study were unlikely to show differences in recalling the early developmental information of their children, and misclassification is unlikely to be differential. The non-differential misclassification can produce biased estimates of odds ratios toward the null value (41), thus the positive outcomes of our study will still exist when considering the parents' reporting errors. Furthermore, the mechanisms underlying infant motor milestones remain poorly characterized according to current literature. The children who did not experience hands-and-knees crawling, but presented bottom shuffling or bunny-hopping during infancy, were excluded from our study as they may have had a confounding effect on our results. Further study is needed to explore the mechanism of these non-typical “crawling behaviors.”

Our findings provided quantitative evidence of the association between early motor development and subsequent motor impairment. Although it is generally not recommended to make any official diagnosis of DCD before 5 years of age (16), it is critical to make early identification of children at higher risk of motor impairment. It is relatively straightforward for parents to notice and report crawling and independent walking onset by daily observation. The findings of the current study will increase our understanding of the early motor development of children with motor impairment, which in turn will have important implications for the early identification of children at higher risk of developing motor impairment in the first 2 years of development. The findings will also further our understanding of the mechanisms underlying motor impairments in children and thereby assist in planning early interventions to support children with motor impairment.

## Conclusion

In conclusion, using a large population-based sample, we found that children's poor motor performance can be observed to diverge from typically developing children by having a delayed crawling onset. Moreover, a delayed walking onset is associated with subsequent motor impairments in all motor domains including fine motor skills. Our results suggested that the transition time from crawling to walking is also associated with later motor impairments, with children with impairments in motor coordination associated significantly with a longer crawling duration before walking compared to typically developing children. Our findings provide important evidence that will help inform practitioners in their early identification of children with motor impairments.

## Data Availability Statement

The original contributions presented in the study are included in the article/Supplementary Material, further inquiries can be directed to the corresponding authors.

## Ethics Statement

The study was approved by the Local Education Board and Ethics Committee of the Children's Hospital of Soochow University. Parental consent and children's assent were obtained before testing. All information acquired was kept confidential and was accessible only to the researchers. Written informed consent to participate in this study was provided by the participants' legal guardian/next of kin.

## Author Contributions

JH conceptualized and designed the study, drafted the initial manuscript, and reviewed and revised the manuscript. GW reviewed and revised the manuscript. JC, MX, and YZ completed the acquisition, analysis, or interpretation of data. HJ and GG designed the data collection instruments, collected data, carried out the initial analyses, and reviewed and revised the manuscript. WD conceptualized and designed the study, drafted the initial manuscript, and reviewed and revised the manuscript. All authors contributed to the article and approved the submitted version.

## Funding

This study was supported by National Natural Science Foundation of China (81673179), National Natural Science Foundation of China (11771146, 11831008, and 81530086), the Science and Technology Commission of Shanghai Municipality (21DZ2202000, 19140903100), Shanghai Municipal Health Commission (2020YJZX0213), Pudong Municipal Health Commission (PW2020D-11), Jiangsu Young Medical Talent Project (QNRC2016250), Gusu Medical Talent Project (GSWS2019026).

## Conflict of Interest

The authors declare that the research was conducted in the absence of any commercial or financial relationships that could be construed as a potential conflict of interest.

## Publisher's Note

All claims expressed in this article are solely those of the authors and do not necessarily represent those of their affiliated organizations, or those of the publisher, the editors and the reviewers. Any product that may be evaluated in this article, or claim that may be made by its manufacturer, is not guaranteed or endorsed by the publisher.
