# Supplementary material for: Early Motor Milestones in Infancy and Later Motor Impairments: A Population-Based Data Linkage Study
Source: Front Psychiatry. 2022 Jan 31;13:809181. doi: 10.3389/fpsyt.2022.809181 (PMC8841506; doi:10.3389/fpsyt.2022.809181)
Supplement: Supplementary file 1 [file Data_Sheet_1.docx]

**TABLE S1 | The total score and subtest scores of MABC-2 by child and family characteristics（n=8395） ^a^**

| Characteristics | Total score  (Mean, SD) | Manual dexterity  (Mean, SD) | Aiming and catching  (Mean, SD) | Balance  (Mean, SD) |
| --- | --- | --- | --- | --- |
| **Child characteristics** |  |  |  |  |
| Children’s age |  |  |  |  |
| 3 | 82.291, 10.314 | 28.665, 5.867 | 18.618, 5.178 | 35.008, 4.333 |
| 4 | 82.751, 10.009 | 28.98, 5.894 | 18.756, 4.938 | 35.014, 4.134 |
| 5 | 82.828, 10.012 | 28.817, 5.779 | 18.964, 5.227 | 35.047, 4.089 |
| 6 | 82.905, 10.213 | 28.993, 5.925 | 18.909, 5.077 | 35.003, 4.323 |
|  |  |  |  |  |
| Gender |  |  |  |  |
| Male | 82.594, 10.184 | 28.799, 5.733 | 18.798, 5.081 | 34.998, 4.264 |
| Female | 82.908, 10.019 | 28.974, 5.938 | 18.892, 5.112 | 35.041, 4.125 |
|  |  |  |  |  |
| BMI |  |  |  |  |
| ≤18 | 82.786, 10/059 | 28.925, 5.843 | 18.859, 5.097 | 35.002, 4.163 |
| >18 | 82.519, 10.521 | 28.527, 5.880 | 18.729, 5.104 | 35.263, 4.476 |
|  |  |  |  |  |
| Right handedness |  |  |  |  |
| No | 82.766, 10.070 | 28.883, 5.843 | 18.853, 5.078 | 35.03, 4.186 |
| Yes | 82.756, 10.672 | 29.155, 5.930 | 18.777, 5.538 | 34.824, 4.229 |
|  |  |  |  |  |
| Eyesight |  |  |  |  |
| Normal | 82.784, 10.127 | 28.899, 5.860 | 18.875, 5.114 | 35.01, 4.214 |
| Abnormal | 82.490, 9.595 | 28.825, 5.657 | 18.464, 4.841 | 35.201, 3.786 |
|  |  |  |  |  |
| Gestational weeks |  |  |  |  |
| <37 | 82.784, 9.285** | 29.297, 5.556 | 19.484, 5.101** | 35.08, 4.118 |
| ≥37 | 82.490, 10.151 | 28.863, 5.868 | 18.8, 5.095 | 35.017, 4.194 |
|  |  |  |  |  |
| Birth weight |  |  |  |  |
| <2500g | 82.445, 9.588 | 28.707, 5.626 | 18.657, 5.137 | 35.081, 4.200 |
| ≥2500g | 82.782, 10.119 | 28.904, 5.858 | 18.859, 5.096 | 35.019, 4.188 |
|  |  |  |  |  |
| **Family characteristics** |  |  |  |  |
| Higher education of mother |  |  |  |  |
| No | 83.200, 9.827*** | 29.155, 5.812*** | 19.011, 5.085** | 35.034, 4.033 |
| Yes | 82.383, 10.310 | 28.665, 5.869 | 18.707, 5.105 | 35.011, 4.321 |
|  |  |  |  |  |
| Higher education of father |  |  |  |  |
| No | 83.210, 9.732** | 29.177, 5.834*** | 18.974, 5.053 | 35.059, 4.040 |
| Yes | 82.480, 10.312 | 28.713, 5.848 | 18.769, 5.125 | 34.998, 4.281 |
|  |  |  |  |  |
| Family annual per-capita income (RMB) ^b^ |  |  |  |  |
| Below | 83.596, 9.955*** | 29.319, 5.734*** | 19.256, 5.081*** | 35.021, 4.102 |
| Above or equal to | 80.306, 10.107 | 27.638, 5.996 | 17.644, 4.955 | 35.023, 4.434 |
|  |  |  |  |  |
| Family structure |  |  |  |  |
| Single families | 83.173, 10.018 | 29.165, 5.289 | 18.937, 5.297 | 35.071, 4.271 |
| Nuclear families | 82.895, 10.026 | 28.958, 5.831 | 18.897, 5.084 | 35.04, 4.157 |
| Extended families | 82.504, 10.224 | 28.763, 5.900 | 18.755, 5.115 | 34.986, 4.245 |
|  |  |  |  |  |
| The number of children in the family |  |  |  |  |
| One | 82.641, 10.141* | 28.845, 5.849 | 18.78, 5.111* | 35.015, 4.195 |
| Two or more | 83.224, 9.911 | 29.075, 5.837 | 19.102, 5.043 | 35.047, 4.164 |
|  |  |  |  |  |
| Maternal age at delivery |  |  |  |  |
| <30 | 82.805, 10.080* | 28.929, 5.834** | 18.868, 5.085 | 35.008, 4.207 |
| 30-34 | 82.129, 10.209 | 28.423, 5.889 | 18.571, 5.094 | 35.135, 4.146 |
| ≥35 | 83.97, 9.945 | 29.652, 5.922 | 19.344, 5.364 | 34.973, 3.886 |
|  |  |  |  |  |
| Maternal complications during pregnancy ^c^ |  |  |  |  |
| No | 82.727, 10.015 | 28.901, 5.826 | 18.816, 5.071 | 35.009, 4.161 |
| Yes | 82.934, 10.431 | 28.867, 5.936 | 18.992, 5.210 | 35.075, 4.303 |

^a^ One-way ANOVA

^b^ The national average family per-capita income of the year before the survey time

^c^ Having one of maternal complications during pregnancy including vaginal bleeding during pregnancy, threatened miscarriage, use of antibiotics, use of fertility drugs, intrauterine distress, fetal asphyxia

**p*<0.05, ***p*<0.01,****p<*0.001

**TABLE S2 | The children’s and family’s characteristics by impairments in gross, fine motor and balance (n =8395****, n%)^a^**

| Characteristic | Manual dexterity | | |  | | Aiming and catching | | | |  |  |  | | Balance | |  | |  |  |  |
| --- | --- | --- | --- | --- | --- | --- | --- | --- | --- | --- | --- | --- | --- | --- | --- | --- | --- | --- | --- | --- |
|  | Significant impairment  (≤5th centile of MABC-2) | At-risk impairment  (6~16th centile of MABC-2) | Typical performance  (>16th centile of MABC-2) | |  | | Significant impairment  (≤5th centile of MABC-2) | At-risk impairment  (6~16th centile of MABC-2) | Typical performance  (>16th centile of MABC-2) | | |  | Significant impairment  (≤5th centile of MABC-2) | | At-risk impairment  (6~16th centile of MABC-2) | | Typical performance  (>16th centile of MABC-2) | |  |  |
| **Child characteristics** |  |  |  | |  | |  |  |  | | |  |  | |  | |  | | |  |
| Children’s age |  |  |  | |  | |  |  |  | | |  |  | |  | |  | | |  |
| 3 | 53(11.2) | 113(11.9) | 744(10.7) | |  | | 83(12.1) | 115(12.0) | 712(10.5) | | |  | 43(10.1) | | 115(11.6) | | 752(10.8) | | |  |
| 4 | 138(29.1) | 281(29.6) | 2140(30.7) | |  | | 208(30.4) | 291(30.3) | 2060(30.5) | | |  | 116(27.3) | | 320(32.4) | | 2123(30.4) | | |  |
| 5 | 160(33.7) | 333(35.1) | 2329(33.4) | |  | | 221(32.3) | 320(33.4) | 2281(33.8) | | |  | 149(35.1) | | 329(33.3) | | 2344(33.6) | | |  |
| 6 | 124(26.1) | 221(23.3) | 1759(25.2) | |  | | 173(25.3) | 233(24.3) | 1698(25.2) | | |  | 117(27.5) | | 225(22.8) | | 1762(25.2) | | |  |
|  |  |  |  | |  | |  |  |  | | |  |  | |  | |  | | |  |
| Gender |  |  |  | |  | |  |  |  | | |  |  | |  | |  | | |  |
| Male | 207(43.6) | 442(46.6) | 3154(45.2) | |  | | 334(48.8) | 429(44.7) | 3040(45) | | |  | 199(46.8) | | 456(46.1) | | 3148(45.1) | | |  |
| Female | 268(56.4) | 506(53.4) | 3818(54.8) | |  | | 351(51.2) | 530(55.3) | 3711(55) | | |  | 226(53.2) | | 533(53.9) | | 3833(54.9) | | |  |
|  |  |  |  | |  | |  |  |  | | |  |  | |  | |  | | |  |
| BMI |  |  |  | |  | |  |  |  | | |  |  | |  | |  | | |  |
| ≤18 | 438(92.2) | 878(92.6) | 6436(92.3) | |  | | 624(91.1) | 885(92.3) | 6243(92.5) | | |  | 387(91.1) | | 922(93.2) | | 6443(92.3) | | |  |
| >18 | 37(7.8) | 70(7.4) | 536(7.7) | |  | | 61(8.9) | 74(7.7) | 508(7.5) | | |  | 38(8.9) | | 67(6.8) | | 538(7.7) | | |  |
|  |  |  |  | |  | |  |  |  | | |  |  | |  | |  | | |  |
| Right handedness |  |  |  | |  | |  |  |  | | |  |  | |  | |  | | |  |
| No | 19(4.0) | 37(3.9) | 299(4.3) | |  | | 34(5.0) | 46(4.8) | 275(4.1) | | |  | 23(5.4) | | 42(4.2) | | 290(4.2) | | |  |
| Yes | 456(96.0) | 911(96.1) | 6673(95.7) | |  | | 651(95.0) | 913(95.2) | 6476(95.9) | | |  | 402(94.6) | | 947(95.8) | | 6691(95.8) | | |  |
|  |  |  |  | |  | |  |  |  | | |  |  | |  | |  | | |  |
| Eyesight |  |  |  | |  | |  |  |  | | |  |  | |  | |  | | |  |
| Normal | 453(95.4) | 875(92.3) | 6541(93.8) | |  | | 640(93.4) | 898(93.6) | 6331(93.8) | | |  | 404(95.1) | | 937(94.7) | | 6528(93.5) | | |  |
| Abnormal | 22(4.6) | 73(7.7) | 431(6.2) | |  | | 45(6.6) | 61(6.4) | 420(6.2) | | |  | 21(4.9) | | 52(5.3) | | 453(6.5) | | |  |
|  |  |  |  | |  | |  |  |  | | |  |  | |  | |  | | |  |
| Gestational weeks |  |  |  | |  | |  |  |  | | |  |  | |  | |  | | |  |
| <37 | 31(6.5) | 66(7.0) | 511(7.3) | |  | | 38(5.5) | 56(5.8) | 514(7.6) | | |  | 34(8.0) | | 65(6.6) | | 509(7.3) | | |  |
| ≥37 | 444(93.5) | 882(93.0) | 6461(92.7) | |  | | 647(94.5) | 903(94.2) | 6237(92.4) | | |  | 391(92.0) | | 924(93.4) | | 6472(92.7) | | |  |
|  |  |  |  | |  | |  |  |  | | |  |  | |  | |  | | |  |
| Birth weight |  |  |  | |  | |  |  |  | | |  |  | |  | |  | | |  |
| <2500g | 27(5.7) | 39(4.1) | 331(4.7) | |  | | 32(4.7) | 50(5.2) | 315(4.7) | | |  | 18(4.2) | | 41(4.1) | | 338(4.8) | | |  |
| ≥2500g | 448(94.3) | 909(95.9) | 6641(95.3) | |  | | 653(95.3) | 909(94.8) | 6436(95.3) | | |  | 407(95.8) | | 948(95.9) | | 6643(95.2) | | |  |
|  |  |  |  | |  | |  |  |  | | |  |  | |  | |  | | |  |
| **Family characteristics** |  |  |  | |  | |  |  |  | | |  |  | |  | |  | | |  |
| Mother has higher education |  |  |  | |  | |  |  |  | | |  |  | |  | |  | | |  |
| No | 205(43.2) | 395(41.7) | 3333(47.8) | |  | | 288(42.0) | 439(45.8) | 3206(47.5) | | |  | 181(42.6) | | 455(46) | | 3297(47.2) | | |  |
| Yes | 270(56.8) | 553(58.3) | 3639(52.2) | |  | | 397(58.0) | 520(54.2) | 3545(52.5) | | |  | 244(57.4) | | 534(54) | | 3684(52.8) | | |  |
|  |  |  |  | |  | |  |  |  | | |  |  | |  | |  | | |  |
| Father has higher education |  |  |  | |  | |  |  |  | | |  |  | |  | |  | | |  |
| No | 166(34.9) | 336(35.4) | 2784(39.9) | |  | | 253(36.9) | 367(38.3) | 2666(39.5) | | |  | 151(35.5) | | 378(38.2) | | 2757(39.5) | | |  |
| Yes | 309(65.1) | 612(64.6) | 4188(60.1) | |  | | 432(63.1) | 592(61.7) | 4085(60.5) | | |  | 274(64.5) | | 611(61.8) | | 4224(60.5) | | |  |
|  |  |  |  | |  | |  |  |  | | |  |  | |  | |  | | |  |
| Family annual per-capita income (RMB) ^a^ |  |  |  | |  | |  |  |  | | |  |  | |  | |  | | |  |
| Below | 288(60.6) | 626(66.0) | 5362(76.9) | |  | | 420(61.3) | 653(68.1) | 5203(77.1) | | |  | 277(65.2) | | 784(79.3) | | 5215(74.7) | | |  |
| Above or equal to | 187(39.4) | 322(34.0) | 1610(23.1) | |  | | 265(38.7) | 306(31.9) | 1548(22.9) | | |  | 148(34.8) | | 205(20.7) | | 1766(25.3) | | |  |
|  |  |  |  | |  | |  |  |  | | |  |  | |  | |  | | |  |
| Family structure |  |  |  | |  | |  |  |  | | |  |  | |  | |  | | |  |
| Single families | 5(1.1) | 13(1.4) | 109(1.6) | |  | | 13(1.9) | 12(1.3) | 102(1.5) | | |  | 4(0.9) | | 15(1.5) | | 108(1.5) | | |  |
| Nuclear families | 303(63.8) | 583(61.5) | 4524(64.9) | |  | | 428(62.5) | 612(63.8) | 4370(64.7) | | |  | 266(62.6) | | 625(63.2) | | 4519(64.7) | | |  |
| Extended families | 167(35.2) | 352(37.1) | 2339(33.5) | |  | | 244(35.6) | 335(34.9) | 2279(33.8) | | |  | 155(36.5) | | 349(35.3) | | 2354(33.7) | | |  |
|  |  |  |  | |  | |  |  |  | | |  |  | |  | |  | | |  |
| The number of children in the family^a^ |  |  |  | |  | |  |  |  | | |  |  | |  | |  | | |  |
| One | 375(78.9) | 758(80.0) | 5463(78.4) | |  | | 554(80.9) | 767(80) | 5275(78.1) | | |  | 337(79.3) | | 790(79.9) | | 5469(78.3) | | |  |
| Two or more | 100(21.1) | 190(20.0) | 1509(21.6) | |  | | 131(19.1) | 192(20) | 1476(21.9) | | |  | 88(20.7) | | 199(20.1) | | 1512(21.7) | | |  |
|  |  |  |  | |  | |  |  |  | | |  |  | |  | |  | | |  |
| Maternal age at delivery |  |  |  | |  | |  |  |  | | |  |  | |  | |  | | |  |
| <30 | 390(82.1) | 801(84.5) | 5906(84.7) | |  | | 569(83.1) | 812(84.7) | 5716(84.7) | | |  | 365(85.9) | | 849(85.8) | | 5883(84.3) | | |  |
| 30-34 | 68(14.3) | 125(13.2) | 806(11.6) | |  | | 94(13.7) | 118(12.3) | 787(11.7) | | |  | 48(11.3) | | 103(10.4) | | 848(12.1) | | |  |
| ≥35 | 17(3.6) | 22(2.3) | 260(3.7) | |  | | 22(3.2) | 29(3) | 248(3.7) | | |  | 12(2.8) | | 37(3.7) | | 250(3.6) | | |  |
|  |  |  |  | |  | |  |  |  | | |  |  | |  | |  | | |  |
| Maternal complications during pregnancy |  |  |  | |  | |  |  |  | | |  |  | |  | |  | | |  |
| No | 382(80.4) | 773(81.5) | 5659(81.2) | |  | | 551(80.4) | 776(80.9) | 5487(81.3) | | |  | 332(78.1) | | 820(82.9) | | 5662(81.1) | | | |
| Yes | 93(19.6) | 175(18.5) | 1313(18.8) | |  | | 134(19.6) | 183(19.1) | 1264(18.7) | | |  | 93(21.9) | | 169(17.1) | | 1319(18.9) | | | |

^a^Pearson chi-square test

^b^The national average family per-capita income of the year before the survey time

^c^Having one of maternal complications during pregnancy including intrauterine distress, asphyxia, cerebral hemorrhage, encephalitis, convulsions and lung diseases

**p*<0.05, ***p*<0.01,****p<*0.001
